# NMR Relaxation Measurements on Complex Samples Based on Real-Time Pure Shift Techniques

**DOI:** 10.3390/molecules25030473

**Published:** 2020-01-22

**Authors:** Xiaoqing Lin, Haolin Zhan, Hong Li, Yuqing Huang, Zhong Chen

**Affiliations:** Department of Electronic Science, Fujian Provincial Key Laboratory of Plasma and Magnetic Resonance, State Key Laboratory for Physical Chemistry of Solid Surfaces, Xiamen University, Xiamen 361005, China; 33320171152756@stu.xmu.edu.cn (X.L.); 33320170155484@stu.xmu.edu.cn (H.Z.); lihong@stu.xmu.edu.cn (H.L.); chenz@xmu.edu.cn (Z.C.)

**Keywords:** longitudinal spin-lattice relaxation (*T*_1_), transverse spin-spin relaxation (*T*_2_), real-time pure shift NMR, Zangger–Sterk (ZS), field inhomogeneity

## Abstract

Longitudinal spin-lattice relaxation (*T*_1_) and transverse spin-spin relaxation (*T*_2_) reveal valuable information for studying molecular dynamics in NMR applications. Accurate relaxation measurements from conventional 1D proton spectra are generally subject to challenges of spectral congestion caused by *J* coupling splittings and spectral line broadenings due to magnetic field inhomogeneity. Here, we present an NMR relaxation method based on real-time pure shift techniques to overcome these two challenges and achieve accurate measurements of *T*_1_ and *T*_2_ relaxation times from complex samples that contain crowded NMR resonances even under inhomogeneous magnetic fields. Both theoretical analyses and detailed experiments are performed to demonstrate the effectiveness and ability of the proposed method for accurate relaxation measurements on complex samples and its practicability to non-ideal magnetic field conditions.

## 1. Introduction

Relaxation times, including longitudinal spin-lattice relaxation (*T*_1_) and transverse spin-spin relaxation (*T*_2_), reveal valuable information for studying molecular dynamics in practical NMR applications [1,2,3]. The combination of relaxation times and other detection parameters, such as chemical shifts and spin-spin scalar (*J*) couplings, endows NMR spectroscopy with its superiority over other detection methods in structural studies [4,5,6]. In detail, *T*_1_ relaxation describes the recovery process of magnetizations along the longitudinal direction after signal excitation. The determination of *T*_1_ relaxation times is useful for acquired signal optimization [7], spin dynamic analysis [8], and chemical exchange evaluation [9]. Whereas, *T*_2_ relaxation reflects the decay of transverse magnetizations caused by spin de-coherence effects, and *T*_2_ relaxation measurements are generally related to applications, such as backbone dynamic detection of proteins [10], chemical reaction monitoring [11], and MRI (Magnetic Resonance Imaging) contrast studies [12]. In general, conventional *T*_1_ and *T*_2_ relaxation measurements in commercial NMR instruments adopt inversion-recovery (IR) [13] and Carr–Purcell Meiboom–Gill (CPMG) sequences [14], respectively. According to a series of acquired signals with their intensities varied as the inversion recovery delay in IR experiments and spin-echo period in CPMG experiments, *T*_1_ and *T*_2_ relaxation times can be calculated by fitting corresponding exponential equations. For simple samples that yield well-resolved resonances in 1D proton (^1^H) NMR, it is easy to assign all peaks according to corresponding chemical shift sites and track their intensity variations. However, accurate relaxation measurements generally encounter the problem of spectral congestion caused by a limited chemical shift range and widespread *J* coupling splittings in 1D ^1^H NMR spectra, particularly for measurements on complex samples that contain numerous protons in different chemical environments. Although *J* couplings between protons provide important molecule structural information, the existence of extensive *J* coupling splittings gives rise to poor signal dispersion in 1D ^1^H NMR spectra along with limited chemical shift distribution, thus hindering accurate relaxation measurements by IR and CPMG experiments. In addition, *J* coupling modulation also leads to signal intensity deviation during the evolution period and influences relaxation measurements for multiplet peaks.

An NMR technique, pure shift NMR spectroscopy [15,16], has been proposed to remove *J* coupling effects and enhance the spectral resolution by converting all multiplets into singlets in the resulting spectra, thus providing a practical solution to the problem of spectral congestion and *J* coupling modulation in NMR relaxation measurements. The pure shift-based method, e.g., Inrec-PSYCHE and CPMG-PSYCHE, [17] as well as PSYCHE-PROJEC*T*_2_ [18], have been implemented for *T*_1_ and *T*_2_ relaxation measurements on complex samples by combining the PSYCHE (Pure Shift Yielded by CHip pulse Excitation) module [19] with relaxation modules, i.e., IR, CPMG, and PROJECT (Periodic Refocusing Of J Evolution by Coherence Transfer) [20] but with a long acquisition time compared to conventional relaxation experiments. A homodecoupled band-selective (HOBS) real-time pure shift technique [21] has been introduced into IR and PROJECT experiments for accelerating pure-shift-based relaxation measurements. Benefitting from real-time acquisition for pure shift extraction in a single scan, HOBS-based relaxation experiments hold the same acquisition efficiency as conventional relaxation experiments [22] but is only suitable for measurements on a particular part of resonances in a single experiment, and multiple experiments are required for all desired resonances.

Besides spectral congestion, magnetic field inhomogeneity, caused by inadequate magnetic field shimming and heterogeneous sample conditions, poses another challenge for accurate relaxation measurements in practical applications. Although signal evolutions during inversion recovery delay in IR experiments for *T*_1_ measurements and spin-echo delay in CPMG experiments for *T*_2_ measurements are free of field inhomogeneity, the acquisition periods in these two relaxation experiments cannot avoid the field inhomogeneity effect, thus giving rise to spectral line broadenings in acquired 1D NMR spectra and hindering accurate relaxation measurements. Recently, a relaxation method based on the combination of intermolecular double-quantum coherence (iDQC) [23] and IR scheme was proposed to achieve *T*_1_ relaxation measurements in inhomogeneous magnetic fields, in which the iDQC scheme is used for eliminating field inhomogeneity and the IR scheme serves for *T*_1_ relaxation evolution [24]. However, *J* coupling splittings still exist and acquired 1D *T*_1_ evolved spectra are presented in absolute-value mode, thus influencing accurate *T*_1_ relaxation calculation.

In view of the possible challenges encountered in relaxation experiments, i.e., spectral congestion caused by *J* coupling splittings and spectral line broadening due to field inhomogeneity, herein we introduce the real-time Zangger–Sterk (ZS) pure shift technique [25] into the relaxation modules of IR and CPMG, and propose an approach, dubbed real-time ZS-IR/CPMG, to overcome these two challenges and achieve accurate *T*_1_ and *T*_2_ relaxation measurements on complex samples containing crowded NMR resonances even under inhomogeneous magnetic fields. Benefitting from the slice-selective property of the ZS pure shift technique [26,27], both *J* coupling splitting and field inhomogeneity primarily along the static field *B*_0_ direction can be eliminated, thus making the real-time ZS-IR/CPMG method able to resolve crowed NMR resonances and record high-resolution 1D spectra for *T*_1_ and *T*_2_ relaxation measurements even in inhomogeneous magnetic fields. Additionally, due to the utilization of the real-time acquisition mode, the real-time ZS-IR/CPMG holds the same acquisition efficiency as conventional relaxation experiments. Theoretical analyses and related experiments were performed to show the effectiveness and applicability of the proposed real-time ZS-IR/CPMG.

## 2. Theories and Methods

**Figure 1:**
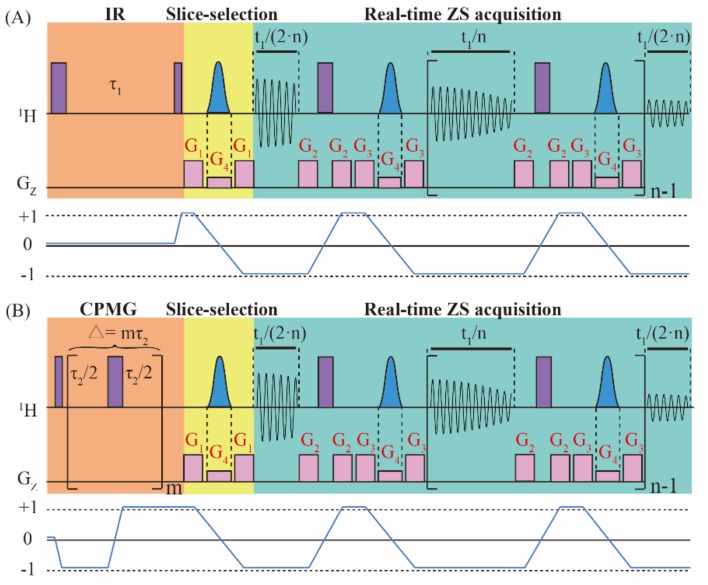
Pulse sequence diagrams of (**A**) real-time ZS-IR and (**B**) real-time ZS-CPMG experiments for measuring *T*_1_ and *T*_2_ relaxation times, respectively. The real-time ZS-IR sequence consists of IR and the real-time ZS module, including slice-selection and real-time ZS acquisition. The real-time ZS-CPMG sequence consists of CPMG and the real-time ZS module. Purple full vertical bars stand for nonselective *π*/2 and *π* pulses, blue Gauss-shaped pulses are Gauss-shaped selective pulses and they are applied with the weak gradients, *G*_4_. *G*_1_, *G*_2_, and *G*_3_ are coherence selection gradients. In the IR module, *τ*_1_ is the incremental inversion recovery delay for the *T*_1_ relaxation evolution process. In the CPMG module, ∆ = *mτ*_2_ is the incremental spin-echo delay for the *T*_2_ relaxation process. In the acquisition period, *t*_1_, acquisition chunks and ZS pure-shift modules are repeated *n* times for extracting pure shift information in a single excitation. The coherence transfer pathways for real-time ZS-IR and real-time ZS-CPMG sequences are given to illustrate the coherence states of spins. Relevant parameters are further defined and explained in the text.

Figure 1 shows the pulse sequence diagram for the real-time ZS-IR/CPMG method, in which the real-time ZS-IR sequence (Figure 1A) aims for *T*_1_ relaxation measurements and the real-time ZS-CPMG sequence (Figure 1B) aims for *T*_2_ relaxation measurements. The design of real-time ZS-IR/CPMG sequences can be considered as the combination of the real-time ZS module with IR and CPMG experiments. The real-time ZS-IR sequence starts with a nonselective *π* pulse to invert longitudinal magnetization into the reverse direction, and *T*_1_ relaxation evolution is recorded in the inversion recovery delay, *τ*_1_. After the *T*_1_ relaxation evolution, a *π*/2 pulse excites the longitudinal magnetization, and the slice-selection part of the real-time ZS module, consisting of a Gauss-shaped selective *π* pulse in union with a weak pulse gradient, *G*_4_, sandwiched by a pair of coherence selective gradients, *G*_1_, is used to select active spins for pure shift evolution before signal acquisition. Then, the real-time ZS acquisition module, including acquisition chunks, nonselective *π* pulses, ZS slice-selection modules of Gauss-shaped selective *π* pulses in union with weak pulse gradients, *G*_4_, and coherence selective gradients, *G*_2_ and *G*_3_, are applied to acquire signals in a single excitation. Acquisition chunks and ZS pure-shift modules are repeated *n* times, in which the first and last acquisition chunks are half of other ones. Finally, 1D pure shift spectra without *J* coupling splitting are recorded for subsequent *T*_1_ relaxation calculation. For the real-time ZS-CPMG sequence, the first nonselective *π*/2 pulse excites the initial magnetization, and then the CPMG module, consisting of *m* repeating spin-echo elements (i.e., *τ*/2-*π*/2-*τ*/2, where *τ* is the interpulse interval), is performed to record *T*_2_ relaxation evolution along with the suppression on field inhomogeneity and *J* coupling modulation. The decay process of *T*_2_ relaxation evolution is achieved by adopting incremental spin-echo delay, ∆ = *mτ*_2_, with different *m*. Similar to the real-time ZS-IR, the real-time ZS module is subsequently used in the real-time ZS-CPMG to acquire 1D pure shift spectra in a single excitation for the following *T*_2_ relaxation calculation.

In order to understand the signal evolution of real-time ZS-IR and real-time ZS-CPMG sequences intuitively, we adopt an AX spin-1/2 weakly coupled system containing *I* and *S* component coupled by a *J*_IS_ coupling. The raising and lowering operator formalism are used to show the theoretical derivation process of the resulting signals. The coherence transfer pathways for signal evolutions are shown in Figure 1. For simplicity, in the following deduction, it is assumed that Ω_I_ represents the chemical shift of the *I* spin in the rotating frame containing intrinsic longitudinal and transverse relaxation times of the *I* spin ($T_{1}^{I}$ and $T_{2}^{I}$). For simplification, the effects of radiation damping, diffusion, and the intermolecular nuclear overhauser effect are omitted in the whole derivation process. Because the *I* and *S* spin in the AX spin-1/2 experience the same evolution, we can only consider the evolution of the *I* spin in the following deduction. Regarding the spin system discussed herein, the reduced operators at the initial thermal equilibrium state with the high-temperature approximation are given as:

(1) $$\sigma_{eq}=I_{Z}$$

where the Bolzmann factor is omitted for simplification, and *I*_Z_ represents the longitudinal magnetization component of *I* spin. For the real-time ZS-IR sequence, the first nonselective *π* pulse converts *I*_Z_ to –*I*_Z_. During longitudinal relaxation evolution, magnetic field inhomogeneity has no effect on the relaxation evolution of the *I* spin and only the intrinsic transverse relaxation rate, $R_{1}^{I}=1/T_{1}^{I}$, is contained. After the evolution of the inversion recovery delay, *τ*_1_, a nonselective *π*/2 pulse flips the magnetization component into the detection direction, and the density operator evolves into $\frac{i}{2}\left(I^{+}-I^{-}\right)\left(1-2e^{-R_{1}^{I}\tau_{1}}\right)$, containing *T*_1_ relaxation evolution. For the real-time ZS-CPMG sequence, the initial reduced density operator, *I*_Z_, is converted into $\frac{i}{2}\left(I^{+}-I^{-}\right)$ by the first nonselective *π*/2 pulse. The CPMG module, consisting of *m* repeating spin-echo elements (*τ*_2_/2-*π*-*τ*_2_/2), would evolve the density operator into $\frac{i}{2}\left(I^{+}-I^{-}\right)e^{-R_{2}^{I}\Delta }$, which contains *T*_2_ relaxation evolution ($R_{2}^{I}=1/T_{2}^{I}$). The effects of field inhomogeneity and the chemical shift are eliminated by spin echo evolution. Additionally, *J* coupling modulation can also be suppressed by setting extremely short *τ*_2_ (i.e., *τ*_2_ << 1/*J*) in the CPMG module. Considering the coherence transfer pathway shown in Figure 1, only the spin term $I^{+}$ is traced in the following deduction, that is:

(2) $$\sigma \left(t\right)=\frac{i}{2}I^{+}R(t)$$

where *R*(*t*) stands for involved relaxation evolution terms. When *t* = *τ*_1_, the relaxation term, $R\left(\tau_{1}\right)=1-2e^{-R_{1}^{I}\tau_{1}}$, is used for the real-time ZS-IR, and when *t* = ∆, the relaxation term, $R\left(\Delta \right)=e^{-R_{2}^{I}\Delta }$, is used for real-time ZS-CPMG. Based on the incremental setting of the inversion recovery delay, *τ*_1_, and spin-echo delay, ∆, the *T*_1_ and *T*_2_ relaxation decay processes are recorded by real-time ZS-IR and real-time ZS-CPMG, respectively.

In the following, the real-time ZS module combined in both real-time ZS-IR and real-time ZS-CPMG is used to recover pure shift spectra for the *T*_1_ and *T*_2_ relaxation calculation. The slice-selection module is first used for filtering out unwanted signals before acquisition. Under the linearly varying magnetic field gradients, *G*_4_, along the z-axis, a small distortion of the nuclear precession frequencies is induced throughout the sample, and the position-dependent frequency shifts to the same resonance in different layers across the effective sample length is added. Therefore, active spins can be manipulated independently by using the frequency-selective Gauss-shaped pulse in each slice depending on the z-coordinate. The combination of the nonselective *π* pulse and the selective Gauss-shaped *π* pulse in union with magnetic field gradients, *G*_4_, can be used to invert the passive spins while leave spin states of the active spins unchanged. The evolved process is:

(3) $$\begin{array}{l} I^{+}R\left(t\right)\overset{\left(2\pi J_{IS}I_{z}S_{z}+\Omega_{I}+\Omega_{S}\right)t_{1}/\left(2\cdot n\right)}{\to }I^{-}R\left(t\right)e^{i\Omega_{I}t_{1}/\left(2\cdot n\right)}\left(\cos A_{J}+2iS_{z}\sin A_{J}\right)\\ \overset{{\left(\pi \right)}_{x}}{\to }I^{+}R\left(t\right)e^{i\Omega_{I}t_{1}/\left(2\cdot n\right)}\left(\cos A_{J}-2iS_{z}\sin A_{J}\right)\\ \overset{{\left(\pi \right)}_{x}^{I}}{\to }I^{-}R\left(t\right)e^{i\Omega_{I}t_{1}/\left(2\cdot n\right)}\left(\cos A_{J}-2iS_{z}\sin A_{J}\right)\\ \overset{\left(2\pi J_{IS}I_{z}S_{z}+\Omega_{I}+\Omega_{S}\right)t_{1}/\left(2\cdot n\right)}{\to }I^{-}R\left(t\right)e^{i\Omega_{I}t_{1}/n} \end{array}$$

where $A_{J}=\pi J_{IS}t_{1}/\left(2\times n\right)$. In order to ensure that *J* couplings are refocused in the middle of each data chunk in the data concatenation, the length of the data chunk is set as 20 ms. The whole acquisition period is performed by repeating *n* times of a single real-time ZS acquisition module. Considering the reduced sample volumes obtained by the slice-selection module, the field inhomogeneity effect along the z-axis can be remarkably reduced during the acquisition part of the real-time ZS module. After the signal acquisition in the acquisition period, *t*_1_, we can obtain the signal as:

(4) $$\sigma \left(t,t_{1}\right)\approx \frac{i}{2}I^{-}e^{i\Omega_{I}t_{1}}R(t)$$

On the basis of Equation (4), the total complex transverse magnetization from *I* spin can be expressed as:

(5) $$\begin{array}{l} M_{+}^{I}\left(t,t_{1}^{\prime }\right)=M_{0}^{I}Tr\left\{I^{+}\sigma \left(t,t_{1}\right)\right\}\\ =\frac{iM_{0}^{I}}{4}e^{i\Omega_{I}t_{1}}R\left(t\right) \end{array}$$

Equation (5) shows a quantitative description for the final real-time ZS-IR and real-time ZS-CPMG signals as a function of the incremental relaxation delay, *t* (*t* = *τ*_1_ or ∆), and incremental variable, *t*_1_. For the acquired 2D data set, the 1D pure chemical shift spectrum is obtained after 1D Fourier transformation over the variable *t*_1_, benefitting for peak recognitions for relaxation calculation from crowded spectra. The relaxation delay, *t*, is relevant to relaxation calculation by the IR and CPMG module, and the *T*_1_ and *T*_2_ relaxation calculations can be obtained by fitting the peak intensity varied with the incremental inversion recovery delay, *τ*_1_, and spin-echo delay, ∆, respectively.

## 3. Experiments

All experiments were performed at 295 K using a Varian 500 MHz NMR spectrometer equipped with a 5 mm ^1^H/^13^C dual-resonance probe having an RF (Radio Frequency) coil of 1.5 cm effective length and a self-shielded z-gradient coil. The magnetic field was well shimmed to a homogeneous condition when performing experiments under a homogeneous magnetic field while the magnetic field was intentionally deshimmed by adjusting the linear shim currents along the z axis. First, a simple sample of 1 M 1-bromobutane (C_4_H_9_Br) dissolved in Chloroform-*d* (CDCl_3_) was used to demonstrate the availability of the real-time ZS-IR and real-time ZS-CPMG methods. Real-time ZS-IR and real-time ZS-CPMG experiments were performed in well-shimmed magnetic field and deshimmed magnetic field with the inhomogeneous line width of 177.35 Hz. Conventional IR and CPMG experiments were performed in well-shimmed magnetic fields. In real-time ZS-IR and real-time ZS-CPMG experiments, parameters relevant to the real-time ZS module were set as following: The durations of the nonselective *π*/2 pulse and Gauss-shaped pulse were 10.35 μs and 16.2 ms. The weak gradients, *G*_4_, matching with the Gauss-shaped pulse were set with an amplitude of 0.56 G/cm and duration of 16.2 ms. The coherence selection gradients, *G*_1_, *G*_2_, and *G*_3_, were set with the amplitudes of 14.65, 9.16, and 9.16 G/cm and the same duration of 0.2 ms. The spectral width was set to 2000 Hz. The number of acquisition chunks was 40 and the acquisition time, *t*_1_, was 0.8s. For the IR module, 10 incremental inversion recovery delays (*τ*_1_ = 0.0625, 0.125, 0.25, 0.5, 1.0, 2.0, 4.0, 8.0, 16.0, 32.0 s) were adopted for tracking *T*_1_ relaxation evolution and the pulse repetition time was 20.0s. For the CPMG module, the interpulse interval, *τ*, was 200μs, 10 incremental spin-echo delays (∆ = 0.025, 0.05, 0.1, 0.2, 0.4, 0.8, 1.6, 3.2, 6.4, 12.8 s) were used for recording *T*_2_ relaxation evolution, and the pulse repetition time was 8.0 s. The scan number was 4, with a total experiment time of 18.7 min for real-time ZS-IR and 8.15 min for real-time ZS-CPMG. For comparison, conventional IR and CPMG experiments were performed with the same incremental inversion recovery delay, *τ*_1_, spin-echo delay, ∆, pulse repetition time, spectral width, and scan number as the real-time ZS-IR and real-time ZS-CPMG experiments, thus with the same experiment times.

Second, experiments on a complex sample of 200 mM quinine (C_20_H_24_N_2_O_2_) in CDCl_3_ were performed to show the advantage of the real-time ZS-IR/CPMG for relaxation measurements on complex samples that contain crowded NMR resonances. Real-time ZS-IR and real-time ZS-CPMG experiments were performed in a well-shimmed magnetic field and deshimmed magnetic field with a line width of 182.23 Hz. Conventional IR and CPMG experiments were performed in well-shimmed magnetic fields. For real-time ZS-IR and real-time ZS-CPMG experiments, the relevant parameters used in the real-time ZS module were set as following: The durations of the nonselective *π*/2 pulse and Gauss-shaped pulse were 10.7 μs and 16.6 ms. The matching weak gradient, *G*_4_, was 0.47 G/cm. The spectral width was 4000 Hz. The number of acquisition chunks was 30 and the acquisition time, *t*_1_, was 0.6 s. The coherence selection gradients, *G*_1_, *G*_2_, and *G*_3_, were set the same as those in the above-mentioned experiments on the simple sample. In the IR module, the incremental inversion recovery delay, *τ*_1_ = 0.04, 0.1, 0.32, 0.84, 1.6, 3.2, 5.0 s, was set for *T*_1_ relaxation evolution. In the CPMG module, the spin-echo delay, ∆ = 0.01, 0.02, 0.04, 0.08, 0.16, 0.32, 0.64, 1.40 s, with the interpulse interval, *τ*_2_, of 200 μs, was used for *T*_2_ relaxation evolution. The scan number was 4, with a total experiment time of 11.05 min for real-time ZS-IR and 5.05 min for real-time ZS-CPMG. For comparison, conventional IR and CPMG experiments were performed with same relaxation parameters i.e. *τ*_1_, ∆, *τ*_2_.

Finally, we carried out real-time ZS-IR/CPMG experiments on a solution of 40 mM azithromycin (C_38_H_72_N_2_O_12_) in CDCl_3_ to further test their applicability on complex practical samples. Experiments were directly performed without any field shimming and locking, introducing a field inhomogeneity of 29.3 Hz. Parameters for the real-time ZS module can be set as following: The matching weak gradient, *G*_4_, was 0.56 G/cm, and the durations of the nonselective *π*/2 pulse and Gauss-shaped pulse were 11.1 μs and 17.1 ms. The spectral width was 2000 Hz. The acquisition time *t*_1_ was set to 0.6 s and the acquisition chunk was 30. The coherence selection gradients, *G*_1_, *G*_2_, and *G*_3_, were the same as those in the above-mentioned experiments on the complex sample. A group of incremental inversion recovery delays (*τ*_1_ = 0.0625, 0.125, 0.25, 0.5, 1.0, 2.5 s) were used in the IR module for *T*_1_ relaxation evolution. A group of spin-echo delays (∆ = 0.01, 0.015, 0.05, 0.09, 0.15, 0.25, 0.4, 0.7 s) was adopted in the CPMG module for *T*_2_ relaxation evolution. The scan number of 4 was used with the total experiment time of 9.23 and 4.98 min for the real-time ZS-IR and real-time ZS-CPMG experiments, respectively.

After data acquisition, the data processing for real-time ZS-IR/CPMG data, including 1D pure shift construction and single exponential fitting, was performed to obtain relaxation values and corresponding measured errors. All data processing is carried out by using a custom-written program on MATLAB 7.8 (MathWorks Natick, MA) and the standard single exponential curve-fitting method built into the VnmrJ software.

## 4. Results and Discussion

### 4.1. A Simple Chemical Solution Sample

**Figure 2:**
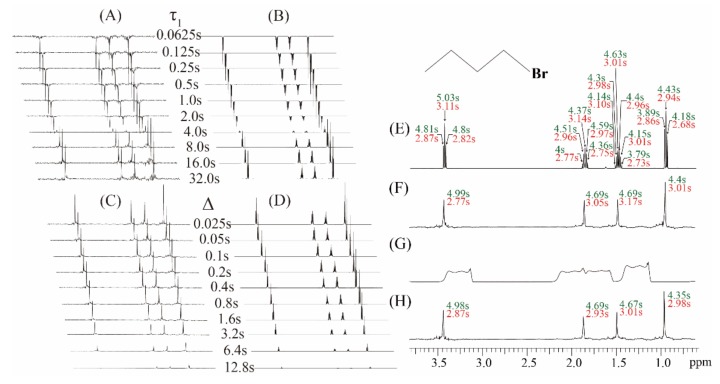
Experiments on the chemical sample of 1 M 1-bromobutane in (C_4_H_9_Br) in Chloroform-*d* (CDCl_3_). (**A**) A group of 1D pure shift spectra by the real-time ZS-IR and (**B**) a group of 1D spectra by the IR, showing the *T*_1_ relaxation evolution as the incremental inversion recovery delay, *τ*_1_, from 0.0625 to 32.0 s. (**C**) A group of 1D pure shift spectra by the real-time ZS-CPMG and (**D**) a group of 1D spectra by the CPMG, showing the *T*_2_ relaxation evolution as the incremental spin-echo delay, ∆, from 0.025 to 12.8 s. (**E**) Calculated *T*_1_ and *T*_2_ values from the conventional IR/CPMG and (**F**) the real-time ZS-IR/CPMG in a well-shimmed magnetic field. (**G**) The 1D spectrum acquired in a deshimmed magnetic field with a 177.35 Hz line width. (**H**) Calculated *T*_1_ and *T*_2_ values from the real-time ZS-IR/CPMG in the deshimmed magnetic field. Molecular structure of 1-bromobutane is shown in the top right of the figure, and all measured *T*_1_ and *T*_2_ are displayed in green and red fonts, respectively.

Experiments on the simple sample of 1-bromobutane were performed to demonstrate the feasibility of the proposed real-time ZS-IR/CPMG. The comparison between conventional IR/CPMG experiments and real-time ZS-IR/CPMG experiments is shown in Figure 2. For *T*_1_ relaxation measurements, two groups of stack spectra evolving as incremental inversion recovery delay, *τ*_1_, from 0.065 to 32.0 s are acquired by the real-time ZS-IR (Figure 2A) and the conventional IR (Figure 2B), respectively. Due to *T*_1_ relaxation evolution in the IR module, peak intensities of these two groups of the stack spectra decay with the incremental inversion recovery delay, *τ*_1_, and corresponding *T*_1_ relaxation calculation were performed by tracking the peak intensity variations. For *T*_2_ relaxation measurements, real-time ZS-CPMG (Figure 2C) and CPMG (Figure 2D) record two groups of array spectra evolving as the incremental spin-echo delay ∆ from 0.025 to 12.8 s, which shows the decay process of *T*_2_ evolution caused by the CPMG module. Similarly, *T*_2_ relaxation values can be calculated by tracking peak intensity variations in these array spectra. Benefitting from pure shift extraction in the real-time ZS-CPMG/IR experiments, all multiplet peaks are simplified into singlet peaks in resulting 1D pure shift spectra (Figure 2A,C), in which all peaks are well-resolved and the standard single exponential curve-fitting method can be directly applied for *T*_2_ calculation. The 1-bromobutane sample contains simple molecular structure and all multiplet peaks are distinctly resolved in the acquired 1D spectra (Figure 2B,D), therefore conventional IR and CPMG experiments can also give satisfactory relaxation results even when *J* coupling splitting exists. Calculated *T*_1_ and *T*_2_ values from conventional IR and CPMG experiments are shown in Figure 2E, and all multiplet peaks caused by *J* couplings among protons give corresponding *T*_1_ and *T*_2_ values marked by green and red font, respectively. Intuitively, each splitting peaks of a given multiplet should give the same relaxation values since they are originated from the identical isotropic proton. However, the relaxation values for the splitting peaks of a given multiplet are generally different because of *J* coupling modulation in the acquisition period. For example, *T*_1_ values of 4.81, 5.03, and 4.80 s and *T*_2_ values of 2.87, 3.11, and 2.82 s are given for the triplet peaks at 3.42 ppm in Figure 2E.

In the real-time ZS-CPMG/IR experiments, relaxation calculation is performed based on 1D pure shift spectra, thus a singlet peak from an isotropic proton has a single *T*_1_ or *T*_2_ value (Figure 2F). For example, the singlet peak that converted from the triplet peak at 3.42 ppm gives 4.99 s for the *T*_1_ value and 2.77 s for the *T*_2_ value, respectively. From the comparison of the calculated relaxation values shown in Figure 2E,F, it can be seen that the calculated *T*_1_ and *T*_2_ values from the real-time ZS-IR/CPMG experiments are similar to those values from the conventional IR/CPMG experiments. All these relaxation measurements are given in Figure 2E,F and performed when the magnetic field is well-shimmed. Unfortunately, when the external magnetic field is degraded, the field inhomogeneity effect would be included into the signal evolution in the acquisition periods of the conventional IR/CPMG experiments and then the recorded 1D spectra would inevitably suffer from inhomogeneous line broadening. For example, the inhomogeneous line width of 177.35 Hz is shown in the 1D spectrum in Figure 2G. From this 1D spectrum, it is clear that all multiplet peaks are covered up by inhomogeneous line broadening, and it is impossible to assign corresponding multiplet peaks for the *T*_1_ and *T*_2_ relaxation measurements by conventional IR/CPMG experiments. In contrast, the real-time ZS-IR/CPMG experiments can effectively eliminate the field inhomogeneity effect based on the reduced sample slice selection by the ZS technique, and high-resolution 1D pure shift spectra can be recovered from inhomogeneous fields for relaxation calculation. Under the same inhomogeneous field shown in Figure 2G, the high-resolution 1D pure shift spectrum along with the calculated *T*_1_ and *T*_2_ values are obtained by the real-time ZS-IR/CPMG experiments (Figure 2H). By using the real-time ZS-IR/CPMG, the *T*_1_ and *T*_2_ values obtained under the inhomogeneous magnetic field are similar to those obtained in the well-shimmed field. Therefore, the feasibility of real-time ZS-IR/CPMG experiments for relaxation measurements without the influence of *J* couplings and field inhomogeneity is self-evident.

### 4.2. Relaxation Measurements on a Complex Sample

**Figure 3:**
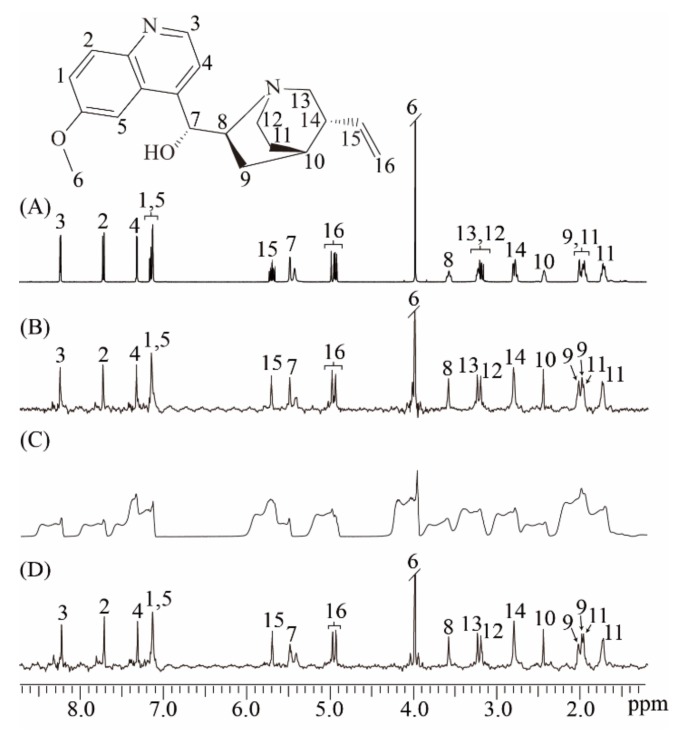
Relaxation measurements for the quinine sample. The molecular structure of quinine with the assigned proton is given at the top. (**A**) Conventional 1D NMR spectrum of the quinine sample in the well-shimmed field. (**B**) 1D pure shift spectrum obtained by the real-time ZS-IR/CPMG in the well-shimmed field. (**C**) Conventional 1D NMR spectrum in the deshimmed field. (**D**) 1D pure shift spectrum by the real-time ZS-IR/CPMG in the deshimmed field.

We performed experiments on the quinine sample in well-shimmed and deshimmed magnetic fields to show the ability of the real-time ZS-IR/CPMG for relaxation measurements on complex samples that contain crowded NMR resonances and suffer from field inhomogeneity (Figure 3). The molecular structure of quinine is given in the top of Figure 3, along with the assigned protons from 1 to 16. [28] A conventional 1D NMR spectrum is acquired in the well-shimmed magnetic field. Numerous multiplet peaks are observed and assigned to corresponding protons (Figure 3A). Although most peaks are well-resolved and identified because of the relatively wide range of the chemical shift distribution, some multiplet peaks remain crowded together, such as multiplet peaks assigned to protons 12 and 13 at around 3.19 ppm. Thus, conventional IR/CPMG experiments based on this 1D spectrum cannot yield accurate relaxation results. Relaxation measurements by the real-time ZS-IR/CPMG experiments are performed based on the pure shift 1D spectrum shown as in Figure 3B. In contrast with the crowded multiplet peaks in the 1D spectrum in Figure 3A, all peaks are simplified into singlets correspondingly assigned to individual protons in the pure shift spectrum in Figure 3B, thus all peaks are presented in a well-resolved manner for the relaxation calculations. For example, two resolved singlet peaks around 3.19 ppm are observed for protons 12 and 13. After the data fitting on the peak intensities of the acquired 1D array pure shift spectra, the *T*_1_/*T*_2_ relaxation values can be easily obtained. When experiments were performed in a deshimmed field, all peaks observed in the conventional 1D spectrum suffer from inhomogeneous line broadening, and it is difficult to distinguish these broadening envelopes along the chemical shift dimension (Figure 3C). Based on this type of 1D spectra, conventional IR/CPMG experiments cannot give correct *T*_1_ and *T*_2_ calculation. However, real-time ZS-IR/CPMG experiments are capable of recording 1D pure shift spectra from an inhomogeneous magnetic field (Figure 3D). Clearly, all peaks observed in the 1D pure shift spectrum acquired in the deshimmed field (Figure 3D) are similar to those acquired in a well-shimmed field (Figure 3B). Therefore, based on the high-resolution 1D pure shift spectrum shown as Figure 3D, *T*_1_ and *T*_2_ relaxation measurements in a deshimmed field can be obtained by real-time ZS-IR/CPMG experiments. To verify the reliability of real-time ZS-IR/CPMG, we also compared the *T*_1_ and *T*_2_ values of singlet peaks measured in conventional IR/CPMG under a well-shimmed magnetic field with those measured in real-time ZS-IR/CPMG. For example, in conventional IR/CPMG experiments, the singlet peak assigned to proton 6 at around 3.10 ppm gives 0.84 s for the *T*_1_ value and 0.58 s for the *T*_2_ value, respectively. In the real-time IR experiment, the measured *T*_1_ result is 0.84 s under a well-shimmed magnetic field and 0.84 s under a deshimmed magnetic field. In the real-time CPMG experiment, the *T*_2_ value for this singlet peak is 0.62 s under a well-shimmed magnetic field and 0.56 s under a deshimmed magnetic field. From the comparison, it can be seen that the calculated *T*_1_ and *T*_2_ values and corresponding errors from conventional IR/CPMG and real-time ZS-IR/CPMG experiments are similar. All calculated *T*_1_ and *T*_2_ values and corresponding errors by real-time ZS-IR/CPMG experiments under well-shimmed and deshimmed field conditions as well as conventional IR/CPMG under well-shimmed conditions are summarized and listed in Table 1.

### 4.3. Relaxation Measurements on a Practical Sample

**Figure 4:**
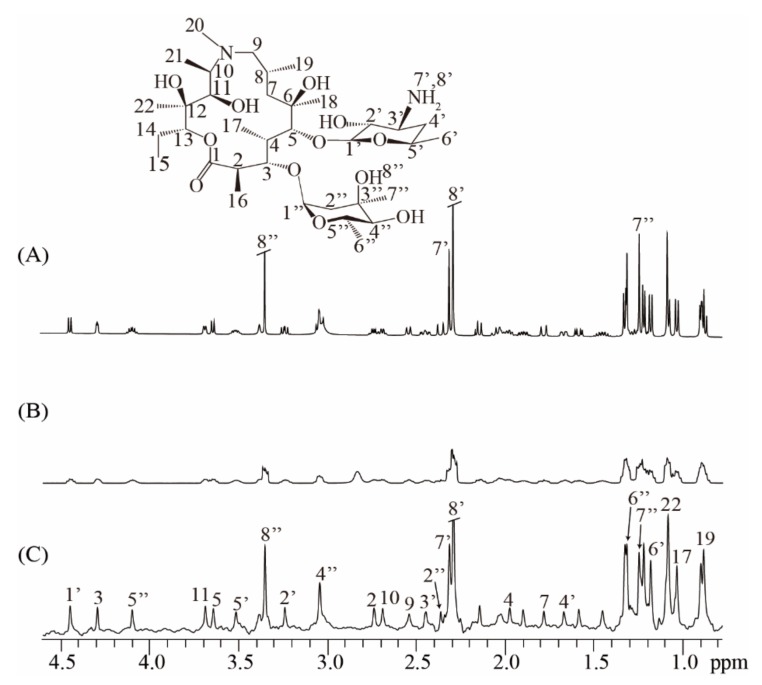
Real-time ZS-IR/CPMG experiments on the practical sample of azithromycin. The molecular structure of azithromycin is at the top. (**A**) Conventional 1D NMR spectrum acquired in homogeneous fields. (**B**) Conventional 1D NMR spectrum directly acquired without the procedure of field shimming and locking, thus suffering from inhomogeneous line broadening of 29.3Hz. (**C**) High-resolution 1D pure shift spectrum obtained by the real-time ZS-IR/CPMG under the same non-ideal field condition as (**B**).

To further show the applicability of the real-time ZS-IR/CPMG on practical samples, we performed *T*_1_ and *T*_2_ relaxation measurements on an antibiotic sample of 40 mM azithromycin (Figure 4). The real-time ZS-IR/CPMG experiments were directly performed under a non-ideal magnetic field condition since the experimental operation of field shimming and locking is ignored. For comparison, a conventional 1D spectrum acquired in the well-shimmed field is presented in Figure 4A, along with the molecular structure of azithromycin. Because of the complex molecular structure in azithromycin [29], a large number of multiplet peaks are observed and crowded in the conventional 1D spectrum, particularly for the spectral region from 0.7 to 2.0 pmm, where peaks suffer from spectral congestion, so they are difficult to assign and separate for further relaxation calculation. This situation becomes even more serious when the field inhomogeneity effect is included. In the conventional 1D spectrum acquired under a non-ideal field condition (Figure 4B), spectral resolution is greatly degraded, and all peaks are broadened and tangled together, making peak assignments and relaxation calculations impossible for conventional IR and CPMG experiments. Nevertheless, the real-time ZS-IR/CPMG provides an effective manner to avoid the field inhomogeneity effect and recover high-resolution 1D pure shift spectra for relaxation measurements. Figure 4C shows the 1D pure shift spectrum acquired in the non-ideal field condition. It is clear that the spectral resolution is significantly enhanced, benefitting from the elimination of field inhomogeneity and *J* coupling splittings, and all singlet peaks marked by 2 to 22, 1′ to 8′, and 2″ to 8″ can be identically assigned to protons given in the molecular structure, even for the congested region between 0.7 and 2.0 ppm. After the relaxation calculation by standard single exponential curve-fitting on peak intensities in the 1D pure shift spectrum, *T*_1_ and *T*_2_ values were obtained by the real-time ZS-IR/CPMG experiments. The relaxation values of singlet peaks were analyzed to further show the accuracy of real-time ZS-IR/CPMG. For example, the *T*_1_ and *T*_2_ values of proton 7′ obtained by conventional IR/CPMG experiments are similar to those values obtained by real-time ZS-IR/CPMG methods. All calculated *T*_1_ and *T*_2_ values and corresponding errors by the real-time ZS-IR/CPMG under this non-ideal magnetic field condition are listed in Table 2. Therefore, the real-time ZS-IR/CPMG provides a useful tool for relaxation measurements on practical complex samples, even under inhomogeneous field conditions where conventional IR/CPMG relaxation methods are not applicable.

*T*_1_ and *T*_2_ values constitute NMR parameters for molecular structure elucidation and dynamic analyses, therefore accurate relaxation measurements are important in practical NMR applications. Because of the limitations in conventional relaxation measurements, i.e., spectral congestion caused by *J* coupling splitting and spectral line broadening caused by non-ideal field conditions, the real-time ZS-IR/CPMG experiments are designed to extract 1D pure shift spectra without the effects of *J* couplings and field inhomogeneity for accurate relaxation measurements. Compared to conventional IR and CPMG, the real-time ZS-IR/CPMG can be applied for measurements on complex samples that contains crowded NMR resonances even under inhomogeneous field conditions. Additionally, the real-time ZS-IR/CPMG adopts an instant acquisition manner by repeating ZS-based decoupling modules during the acquisition period to record 1D pure shift spectra in a single excitation, avoiding the pseudo-2D acquisition that is commonly used in standard pure shift NMR. Therefore, the experimental time of real-time ZS-IR/CPMG experiments remains similar to that of conventional IR/CPMG experiments, i.e., depending on the incremental evolution of spin echo and inversion recovery delays. Although the instant acquisition can accelerate real-time ZS-IR/CPMG experiments, it inevitably introduces undesired artefacts in reconstructed pure shift spectra, thus leading to incorrect peak assignments. In addition, these artifacts introduced by pure shift experiments may degrade the spectral quality. For example, the sinusoid distortion of the baseline shown in the real-time ZS-IR/CPMG experiments on quinine (Figure 3) and azithromycin (Figure 4) are generally caused by chunking sidebands (or periodic artifacts) and residual *J* coupling modulation. The occurrence of sinusoidal distortion of the baseline would have an impact on the relaxation fitting process, which is why the calculated relaxation values by real-time ZS-CPMG/IR experiments slightly different from those by standard CPMG/IR. However, in real-time ZS-CPMG/IR experiments, relaxation values are calculated by fitting the peak intensity via the single exponential curve-fitting, therefore this impact is generally not critical and it is also related to the sample concentration. For high-concentration simple samples, such as 1-bromobutane, there are fewer artifacts in the resulting pure shift spectra, then the influence of the sinusoidal distortion of the baseline on the fitting results can be ignored. For complex samples with a lower concentration, such as quinine and azithromycin, the influence can be obviously observed, but satisfactory calculated relaxation results are still obtained. Moreover, some special data processing, such as deep learning techniques [30], may be suitable for suppressing these undesired signals and recovering clean pure shift spectra for relaxation calculations. Additionally, due to the reduced sample slices adopted in the ZS-based pure shift scheme, real-time ZS-IR/CPMG experiments intrinsically suffer from low signal intensity. In general, signal intensities in real-time ZS-IR/CPMG experiments are approximately 5% to 10% of that in conventional IR/CPMG experiments. Despite all this, our experiments on the azithromycin sample with only 40 mM suggest that real-time ZS-IR/CPMG remains suitable for detection on samples containing a relatively low concentration with dozens of millimoles. A multiband excitation strategy [31,32] may be further introduced into real-time ZS-IR/CPMG experiments to compensate the signal loss caused by the ZS-based scheme.

It is worth mentioning that the diffusion coefficient is one of the molecular dynamic information of interest in NMR applications, and its measurement process is similar to the single exponential fitting of relaxation measurements. By fitting the variable of incremental diffusion gradients, 2D DOSY (Diffusion-Order NMR SpectroscopY) [33,34] correlated chemical shift information with diffusion coefficients can be obtained. In practical measurements on complex mixtures, DOSY experiments are generally limited by the spectral resolution along the chemical shift dimension. Therefore, the pure shift technique is also exploited in the DOSY experiment for further improving the spectral resolution along the chemical shift dimension by converting multiplet peaks into singlets, and some pure shift-based DOSY experiments are proposed for DOSY measurements on complex samples, such as ZS-DOSY [35], PSYCHE-iDOSY [36], and PSDE-DOSY [37] experiments. These DOSY experiments are performed based on pseudo-3D acquisition, inevitably requiring a long acquisition time. Similarly, conventional relaxation measurements also encounter spectral congestions on complex samples. Thus, we proposed the real-time IR/CPMG method by introducing the real-time ZS pure shift technique into IR and CPMG for relaxation measurements on complex samples containing crowded NMR resonances even under inhomogeneous magnetic fields. Benefitting from the utilization of the real-time acquisition mode, the real-time ZS-IR/CPMG experiments only acquire 2D acquisition and hold the same acquisition efficiency as conventional relaxation experiments.

## 5. Conclusions

In this study, we presented an NMR relaxation method, real-time ZS-IR/CPMG, based on the combination of the real-time ZS pure shift technique with IR and CPMG relaxation schemes, for *T*_1_ and *T*_2_ relaxation measurements on complex samples even under inhomogeneous magnetic field conditions. The proposed method can avoid two major challenges in conventional IR/CPMG experiments, i.e., spectral congestion caused by *J* coupling splitting and spectral line broadening originating from field inhomogeneity primarily along the static field *B*_0_ direction, thus achieving accurate relaxation measurements. Experiments on three types of samples, a simple chemical solution of 1-bromobutane, a complex sample of quinine, and a real sample of azithromycin, were performed to show the ability of the real-time ZS-IR/CPMG for acquiring *T*_1_/*T*_2_ relaxation information from crowded and broadening NMR resonances. The real-time ZS-IR/CPMG may provide an effective and complementary tool to conventional IR/CPMG for relaxation measurements with extensive applications to complex sample systems or even under non-ideal magnetic field conditions.

## Figures and Tables

**Table 1:** *T*_2_ relation values and errors of some typical peaks by real-time ZS-IR/CPMG and conventional IR/CPMG.

Proton	Chemical Shift (ppm)	*T*_1_ by Real-Time ZS-IR in Well-Shimmed/Deshimmed Fields (s)	*T*_2_ by Real-time ZS-CPMG in Well-Shimmed/Deshimmed Fields (s)
1,5	6.45	1.36 ± 0.03/1.32 ± 0.05	0.87 ± 0.02/0.85 ± 0.02
2	7.06	1.84 ± 0.07/1.80 ± 0.06	1.08 ± 0.04/1.09 ± 0.03
3	7.60	1.69 ± 0.08/1.66 ± 0.06	0.89 ± 0.04/0.90 ± 0.02
4	6.65	1.05 ± 0.01/1.03 ± 0.04	0.67 ± 0.02/0.65 ± 0.02
6	3.10	0.84 ± 0.01/0.84 ± 0.01 0.84 ± 0.01*	0.62 ± 0.02/0.56 ± 0.01 0.58 ± 0.01*
7	4.70	0.54 ± 0.02/0.53 ± 0.02 0.52 ± 0.01*	0.12 ± 0.01/0.13 ± 0.01 0.16 ± 0.01*
8	2.68	0.38 ± 0.01/0.40 ± 0.01	0.22 ± 0.01/0.22 ± 0.01
9	1.00 0.96	0.66 ± 0.01/0.66 ± 0.04 0.42 ± 0.01/0.45 ± 0.01	0.31 ± 0.01/0.36 ± 0.01 0.26 ± 0.01/0.30 ± 0.01
10	1.46	0.83 ± 0.01/0.84 ± 0.01	0.36 ± 0.01/0.36 ± 0.01
11	0.94 0.69	0.42 ± 0.01/0.43 ± 0.01 0.39 ± 0.01/0.40 ± 0.01	0.26 ± 0.01/0.25 ± 0.01 0.24 ± 0.01/0.24 ± 0.01
12	2.26	0.43 ± 0.01/0.44 ± 0.01	0.28 ± 0.01/0.28 ± 0.01
13	2.29	0.57 ± 0.01/0.58 ± 0.01	0.31 ± 0.01/0.30 ± 0.01
14	1.83	0.40 ± 0.01/0.41 ± 0.01	0.26 ± 0.01/0.24 ± 0.01
15	4.92	1.45 ± 0.04/1.39 ± 0.01	0.73 ± 0.01/0.71 ± 0.01
16	4.15 4.11	1.24 ± 0.01/1.25 ± 0.02 1.30 ± 0.01/1.34 ± 0.02	0.73 ± 0.01/0.71 ± 0.01 0.71 ± 0.02/0.69 ± 0.02

The asterisk * represents measured relaxation results obtained by conventional IR or CPMG experiments under a well-shimmed magnetic field.

**Table 2:** *T*_2_ relaxation values and errors of some typical peaks of azithromycin by Real-time ZS-IR/CPMG under non-ideal field condition and conventional IR/CPMG under a well-shimmed magnetic field.

Proton	Chemical Shift (ppm)	*T*_1_ Relaxation (s)	*T*_2_ Relaxation (s)
2	2.76	0.62 ± 0.03	0.40 ± 0.05
3	4.30	0.48 ± 0.02	0.33 ± 0.03
4	1.99	0.39 ± 0.01	0.30 ± 0.03
5	3.65	0.41 ± 0.01	0.30 ± 0.03
7	1.80	0.37 ± 0.02	0.21 ± 0.02
9	2.55	0.28 ± 0.02	0.22 ± 0.02
10	2.70	0.39 ± 0.02	0.26 ± 0.03
11	3.70	0.37 ± 0.02	0.23 ± 0.02
17	1.05	0.36 ± 0.02	0.29 ± 0.02
19	0.92	0.68 ± 0.03	0.45 ± 0.04
22	1.09	0.33 ± 0.01	0.28 ± 0.01
1′	4.47	0.44 ± 0.02	0.31 ± 0.06
2′	3.23	0.70 ± 0.03	0.33 ± 0.03
3′	2.48	0.51 ± 0.05	0.26 ± 0.03
4′	1.69	0.36 ± 0.02	0.22 ± 0.01
5′	3.52	0.51 ± 0.05	0.29 ± 0.01
6′	1.21	0.40 ± 0.01	0.32 ± 0.01
7′	2.32	0.43 ± 0.01 0.43 ± 0.01*	0.31 ± 0.01 0.32 ± 0.01*
8′	2.30	0.41 ± 0.01 0.41 ± 0.01*	0.28 ± 0.01 0.30 ± 0.01*
2″	2.39	0.36 ± 0.02	0.26 ± 0.01
4″	2.07	0.79 ± 0.01	0.34 ± 0.03
5″	4.12	0.49 ± 0.02	0.31 ± 0.05
6″	1.34	0.45 ± 0.02	0.32 ± 0.01
7″	1.27	0.46 ± 0.01 0.45 ± 0.01*	0.32 ± 0.02 0.35 ± 0.01*
8″	3.34	0.67 ± 0.01 0.66 ± 0.01*	0.40 ± 0.01 0.45 ± 0.01*

The asterisk * represents measured relaxation results obtained by conventional IR or CPMG experiments under a well-shimmed magnetic field.
